# Startling Suggestion

**Published:** 1920-07-17

**Authors:** 


### Startling Suggestion.

### Surprising Category of Dangerous Foods.

A DEFINITE expression of opinion from a responsible public health official that milk, sugar, and even white flour may be dangerous foods is a startling affair. If we are told one day that the open grate is unhygienic, we lift our eyebrows in mild astonishment, gather closer round the hearth in these raw July evenings, and nearly forget all about it. But a proposition, equally serious, that perhaps

**THE PUBLIC WELL-BEING**—(*continued*)

milk, sugar, and white flour are positively dangerous to health, after all these years of propaganda in favour of milk and sugar as prime health-building properties, is an affair too surprising to be passed over by the lift of an eyebrow.

Yet in a closely reasoned and obviously well-considered statement by Dr. James Wheatley, medical officer of health for Shropshire, before the Central Counties' Branch of the British Dental Association, this deduction was logically drawn. He was dealing with the almost universal extent of dental caries, its cause and prevention, and in the course of a most interesting analysis of the subject he inclined to the view that the causation of the disease was dependent on food. One of his strongest points was that in the county of Salop, children entering school at the age of five averaged, before the war, six decayed teeth per child. This was an appalling state of things. But since the war, children in that category of age have shown the most remarkable improvement, the number free from caries having increased from five to 44.4 per cent. If these figures were applicable to the whole of the school population of the country, there should now be 100,000 fewer carious teeth among school children than in 1914. The fact to be borne in mind about these comparisons is that children aged five have been subjected to war conditions during the whole of their lives.

War Experience.

Dr. Wheatley attributes the decrease to the restriction and modification of food during the war, and secondarily to the energetic educational campaign by health visitors, medical officers and teachers. He agreed it was difficult to apportion the effect on the teeth of the various alterations of food, but it seemed likely that the wholesale cutting down of sugar and the almost total elimination of sweets were the most powerful factors. It seemed likely, too, that the alteration in the character of the bread had also a considerable effect. "Before the war," added Dr. Wheatley, "it was almost the rule for children to throw away their crusts or get rid of them in some other way. During the war it was the rarest thing to see crusts thrown away. I have no doubt whatever that owing to these various changes better habits of mastication were formed, with good results to the teeth." The diminished consumption of milk might also have been a contributing factor, as there could be little doubt that milk, taken particularly the last thing at night without any food requiring mastication, was one of the most powerful means of causing dental caries. In childhood there was an excess of milk and sweets, a sloppy diet, frequent meals, and often a glass of milk and a biscuit or a sweet on retiring. Where these conditions were absent there was no excess decay. The problem, therefore, was a dietetic one. The Ministry of Health should be urged to place the cause and prevention of dental caries in the forefront of their scheme of research work in order to clear up points of doubt, and so be in a position to direct a national campaign with real authority. Caries was the cause of an enormous amount of illness, but it was essentially preventable.

Inquiry Proposed.

The Medical Officer of Health for Birmingham (Dr. Robertson) said he had felt for years that some definite investigation and pronouncement on the subject of dental caries was required from the Government. If it were true that substances like milk and sugar, and, perhaps, white flour, were among the most dangerous foods we could use, it would take very strong evidence to prove their harmfulness because they could not easily be induced to give them up. The medical profession had believed in these things for generations. He hoped the strongest possible pressure would be brought upon the Government to institute an enquiry into the subject, to ascertain what the principal causes of dental caries were, and to decide upon the best method of prevention. The number of tuberculous cases in which the mastication of food was prevented through decayed teeth was perfectly appalling.

Dr. Auden, Medical Officer to the Birmingham Education Authority, also spoke on the effect of decayed teeth on the health of the community. He mentioned that in the cases of 24,000 school children examined in Birmingham 49,000 cases had to be extracted as beyond saving.

